# Impact of dietary *Chlorella vulgaris* and carbohydrate-active enzymes incorporation on plasma metabolites and liver lipid composition of broilers

**DOI:** 10.1186/s12917-021-02932-8

**Published:** 2021-06-29

**Authors:** Diogo Francisco Maurício Coelho, Cristina Maria Riscado Pereira Mateus Alfaia, José Miguel Pestana Assunção, Mónica Costa, Rui Manuel Amaro Pinto, Carlos Mendes Godinho de Andrade Fontes, Madalena M. Lordelo, José António Mestre Prates

**Affiliations:** 1 CIISA - Centro de Investigação Interdisciplinar em Sanidade Animal, Faculdade de Medicina Veterinária, Universidade de Lisboa, 1300-477 Lisboa, Portugal; 2 iMed.UL, Faculdade de Farmácia, Universidade de Lisboa, Avenida Professor Gama Pinto, 1649-003 Lisboa, Portugal; 3 LEAF - Linking Landscape, Environment, Agriculture And Food, Instituto Superior de Agronomia, Universidade de Lisboa, Tapada da Ajuda, 1349-017 Lisboa, Portugal

**Keywords:** *Chlorella vulgaris*, CAZymes, plasma metabolites, liver composition, broiler

## Abstract

**Background:**

*Chlorella vulgaris* has been proposed as a sustainable green feedstock in poultry nutrition due to its ease of cultivation, minimal environmental impact and balanced nutritional composition. However, the majority of studies documents the use of *C. vulgaris* as a dietary supplement in broilers instead of a feed ingredient. To the best of our knowledge, no report has shown the effect of a high-level incorporation (>2 % in the diet) of *C. vulgaris* on plasma metabolites and hepatic lipid composition of broilers. One hundred and twenty Ross 308 male birds were housed in 40 wired-floor cages and randomly distributed by the following experimental diets at 22 days of age (*n* = 10) during 15 days: (1) a corn-soybean meal based diet (control); (2) based diet with 10% of *C. vulgaris*; (3) diet *2* supplemented with 0.005% Rovabio^®^ Excel AP; and (4) diet *2* supplemented with 0.01% of a pre-selected four-CAZyme mixture.

**Results:**

The inclusion of *C. vulgaris* at 10% in the diet, regardless of the presence of exogenous CAZymes, changed plasma metabolites but did not compromise broilers growth. Plasma total lipids increased in broilers fed *C. vulgaris* combined with the two feed CAZymes (*p* < 0.001) compared with the control diet. Moreover, the supplementation with Rovabio^®^ increased total cholesterol and LDL-cholesterol, while the addition of the four-CAZyme mixture increased triacylglycerols, VLDL-cholesterol and ALP activity. In opposition, HDL-cholesterol levels decreased in broilers fed microalga alone (*p* = 0.002). Regarding hepatic composition, the inclusion of *C. vulgaris* in broiler diets, individually or combined with exogenous CAZymes, had a minor effect on fatty acids but improved the *n*-6/*n*-3 ratio and total carotenoids.

**Conclusions:**

In summary, the inclusion of a high level (10%) of *C. vulgaris* in broiler´s diet, regardless of the presence of exogenous CAZymes, improved hepatic antioxidant composition and did not impair broiler’s performance. In addition, the feed supplementation with CAZymes increased broilers lipemia. Therefore, dietary *C. vulgaris* at this incorporation level seems to be safe for animal health and do not compromise performance traits, with no need of CAZymes supplementation.

**Supplementary Information:**

The online version contains supplementary material available at 10.1186/s12917-021-02932-8.

## Background

Broiler meat is one of the most consumed meats worldwide, thus being a major source of animal protein for human consumption [1]. The increasing demand of broiler meat has brought new challenges to livestock agriculture. In addition, the increase of broiler production led to an intensive production of conventional feed raw materials, mainly corn and soybean, which has a negative impact on environmental sustainability. Moreover, health-conscious consumers are driving the demand for products with a high nutritional value [2, 3].

The use of microalgae as animal feed, mainly in poultry, has been considered a sustainable and promising alternative to face the challenge imposed on livestock agriculture. Microalgae production does not require arable land or potable water, thus not competing with human food. Additionally, through the photosynthetic process, microalgae could help mitigate the increase of atmospheric carbon dioxide [4]. Moreover, microalgae display an interesting nutritional composition, with a balanced protein concentration and amino acid profile, which is comparable or superior to the conventional protein sources used in animal feeding. Microalgae also present interesting contents of *n*-3 and *n*-6 polyunsaturated fatty acids (PUFA), including eicosapentaenoic acid (EPA) and docosahexaenoic acid (DHA), carbohydrates, vitamins, minerals, carotenoids and antioxidants [5, 6]. *Chlorella vulgaris*, a freshwater eukaryotic green microalga, stands out for its relative ease of cultivation, high yield of biomass production and a well-balanced nutritional composition, being one of the most produced microalgae worldwide [7, 8]. However, *C. vulgaris* contains a recalcitrant cell wall, composed by a diverse and complex matrix of cross-linked insoluble carbohydrates, which are largely indigestible by monogastrics [9–11]. Carbohydrate-Active enZymes (CAZymes) have been applied in monogastric livestock as feed additives [12, 13]. Beyond its recognized ability to degrade cereal cell walls [14], CAZymes also demonstrated the capacity to disrupt microalgae cell walls [15, 16]. Recently, a recombinant four-CAZyme mixture, was developed *in vitro* to partially degrade the *C. vulgaris*, enabling the release of trapped nutrients [17]. Furthermore, *C. vulgaris* has been regarded as dietary supplement in human and animal studies due to its antioxidant, antidiabetic, antihyperlipidemic, immunomodulatory and anti-inflammatory properties [18, 19]. Thus, the incorporation of *C. vulgaris* not only improves the nutritional value of diets, and consequently growth performance, but also enhances animal health [20]. However, until to date, the majority of these studies have been carried out with the incorporation of *C. vulgaris* at low levels (<2 % in diet). Hereupon, this study was conducted to assess the effect of dietary *C. vulgaris* incorporation at 10%, supplemented or not with the commercially available Rovabio^®^ and the four-CAZyme mixture described by Coelho et al. [17], on plasma metabolites and hepatic lipid composition of broilers.

## Results

### Feed Intake and growth performance of broilers

Table 1 displays the results on feed intake and growth performance of broilers for contextualization purposes. The experimental diets had no significant effect neither on feed intake nor on growth performance (*p* > 0.05). Values of average daily feed intake (ADFI), average daily gain (ADG) and feed conversion ratio (FCR) were 127 g, 80.5 and 1.56 g, respectively.

**Table 1:** Effect of experimental diets on growth performance parameters of broilers

Item	Control	CH	CHR	CHM	SEM	*p*-value
Initial weight, g	786.8	788.3	780.2	783.4	12.67	0.969
Final weight, g	1867.4	1927.8	1923.2	1929.2	52.89	0.811
ADG, g/d	77.2	81.4	81.6	81.8	2.406	0.991
ADFI, g/pen	128.4	124.0	124.4	131.1	3.644	0.464
FCR	1.590	1.537	1.528	1.602	0.037	0.395

The broilers were fed: (1) a corn-soybean based diet (Control); (2) the based diet plus 10 % *C. vulgaris* (CH); (3) diet 2 supplemented with 0.005 % Rovabio^®^ Excel AP; and (4) diet 2 supplemented with 0.01 % of a pre-selected four-CAZyme mixture (CHM)

SEM - standard error of the mean; ADFI - average daily feed intake; ADG - average daily weight gain; FCR - feed conversion ratio

### Plasma biochemical profile

Plasma metabolites of broilers fed 10 % of *C. vulgaris*, individually or combined with exogenous CAZymes, are presented in Table 2. Total lipids were significantly increased (*p* < 0.001) in broilers fed with CHR and CHM diets when compared with the ones fed CH and control diets. Broilers fed CHR had higher total cholesterol (*p* < 0.001) and low-density lipoprotein cholesterol (LDL) (*p* < 0.001) relative to the other diets. CHM had higher triacylglycerols (TAG) (*p* < 0.001) and very-low-density lipoprotein cholesterol (VLDL) (*p* < 0.001) when compared to CH and control diets. On the contrary, CH diet decreased high-density lipoprotein cholesterol (HDL) levels (*p* = 0.002). These alterations resulted in a lower total cholesterol/HDL-cholesterol ratio (*p* < 0.001), simultaneously with lower total protein, in the control diet compared to the other diets. In addition, broilers fed CHM diet decreased glucose (*p* = 0.001) when compared to the control diet. Although creatinine levels remained unchanged among experimental diets (p > 0.05), urea increased (*p* < 0.001) in broilers fed *C. vulgaris* alone.

**Table 2:** Effect of experimental diets on plasma metabolites of broilers

Item	Control	CH	CHR	CHM	SEM	*p*-value
***Plasma metabolites***						
Total lipids (mg/L)^1^	3578^a^	3500^a^	3941^b^	3932^b^	60.26	< 0.001
TAG (mg/L)	358^a^	402^b^	475^bc^	526^c^	23.68	< 0.001
Total cholesterol (mg/L)	860^a^	799^a^	983^c^	953^b^	23.36	< 0.001
HDL-cholesterol (mg/L)	641^b^	566^a^	641^b^	672^b^	18.78	0.002
LDL-cholesterol (mg/L)	125^a^	150^b^	240^d^	178^c^	4.63	< 0.001
VLDL-cholesterol (mg/L)^2^	71.6^a^	80.4^ab^	95.0^bc^	105^c^	4.73	< 0.001
Total cholesterol/HDL-C	1.34^a^	1.41^b^	1.54^c^	1.42^b^	0.017	< 0.001
Glucose (mg/L)	2571^b^	2463^ab^	2452^ab^	2357^a^	33.78	0.001
Urea (mg/L)	10.80^ab^	15.80^c^	13.70^bc^	9.60^a^	0.83	< 0.001
Creatinine (mg/L)	0.15	0.13	0.09	0.16	0.02	0.160
Total protein (g/L)	25.87^a^	28.93^b^	28.97^b^	28.85^b^	0.68	0.005
***Plasma hepatic markers***						
ALT (U/L)	3.70	4.60	5.50	5.30	0.589	0.146
AST (U/L)	235.7	297.2	314.0	241.4	23.7	0.056
ALP (U/L)	3552^b^	2820^ab^	2149^a^	5040^c^	198.6	< 0.001
GGT (U/L)	21.2^b^	19.4^b^	20.5^b^	15.3^a^	0.920	< 0.001

The broilers were fed: (1) a corn-soybean based diet (Control); (2) the based diet plus 10% *C. vulgaris* (CH); (3) diet 2 supplemented with 0.005% Rovabio^®^ Excel AP; and (4) diet 2 supplemented with 0.01% of a pre-selected four-CAZyme mixture (CHM)

SEM - standard error of the mean; TAG - triacylglycerols; HDL - high-density lipoproteins; LDL - low-density lipoproteins; VLDL - very low-density lipoproteins; ALT - alanine aminotransferase (EC 2.6.1.2); AST - aspartate aminotransferase (E.C. 2.6.1.1); ALP - alkaline phosphatase (EC 3.1.3.1); GGT - gamma-glutamyltransferase (EC 2.3.2.13)

^1^ Total lipids = [total cholesterol] × 1.12 + [TAG] × 1.33 + 148, as described by Covaci et al. [21]

^2^ VLDL-cholesterol = 1/5 [TAG], as described by Friedewald et al. [22]

^a.b,c^ Different superscripts within a row indicate a significant difference (*p* < 0.05)

Regarding liver enzymes, alanine aminotransferase (ALT) and aspartate aminotransferase (AST) activities in plasma were unaffected (*p* > 0.05) by the experimental diets. Curiously, CHM diet increased plasma alkaline phosphatase (ALP) (*p* < 0.001) but decreased gamma-glutamyltransferase (GGT) (*p* < 0.001), when compared to the other diets.

### Hepatic total lipids, total cholesterol and fatty acid composition

The effect of *C. vulgaris*, individually or in combination with feed enzymes, on hepatic total lipids, cholesterol content and fatty acid composition of broilers is shown in Table 3. Experimental diets did not contribute to significant differences on total lipids and total cholesterol contents (*p* > 0.05). The predominant fatty acids found in liver were C18:0 (22.9–24.4 %), C18:2*n*-6 (20.0-20.6 %), C16:0 (17.6–19.6 %), C20:4*n*-6 (11.5–13.1 %) and C18:1*c*9 (10.1–12.9 % of total FAME). However, only 3 minor fatty acids out of the 33 fatty acids identified were affected by the experimental diets. The proportion of C16:1c7 (*p* < 0.001) was higher in broilers fed microalga, with and without CAZymes, compared to those fed the control diet. Similarly, the proportion of C20:3*n*-3 was significantly lower in the control diet relative to CH (*p* = 0.032). Moreover, the experimental diets did not change the partial sums of fatty acids and the PUFA/SFA ratio (*p* > 0.05), but a significant decrease of *n*-6/*n*-3 ratio was observed in all microalga diets compared to the control diet (*p* = 0.025).

**Table 3:** Effect of experimental diets on hepatic total lipids, total cholesterol and fatty acid (FA) composition of broilers

Item	Control	CH	CHR	CHM	SEM	*p*-value
***Total lipids, g/100 g***	3.10	2.90	3.20	2.98	0.126	0.363
***Total cholesterol, mg/g***	1.09	1.17	1.18	1.06	0.073	0.614
***FA composition, g/100 g FA***						
C10:0	0.01	0.00	0.01	0.00	0.002	0.192
C12:0	0.01	0.01	0.01	0.01	0.003	0.446
C14:0	0.22	0.17	0.22	0.20	0.025	0.395
C14:1*c*9	0.02	0.01	0.01	0.01	0.005	0.851
C15:0	0.05	0.05	0.05	0.05	0.005	0.988
DMA-C16:0	0.11	0.13	0.13	0.11	0.018	0.819
C16:0	19.6	17.6	18.6	18.6	0.920	0.533
C16:1*c*7	0.18^a^	0.28^b^	0.27^b^	0.30^b^	0.014	< 0.001
C16:1*c*9	0.69	0.46	0.48	0.62	0.135	0.580
C17:0	0.34	0.41	0.41	0.40	0.028	0.210
C17:1*c*9	0.03	0.04	0.04	0.04	0.005	0.108
DMA-C18:0	0.18	0.26	0.26	0.21	0.037	0.369
DMA-C18:1	0.02	0.01	0.02	0.02	0.004	0.848
C18:0	22.9	24.4	24.3	23.6	0.540	0.222
C18:1*c*9	12.9	10.1	10.5	12.2	1.628	0.562
C18:1*c*11	1.11	1.26	1.22	1.28	0.044	0.048
C18:2*n*-6	20.0	20.5	20.6	20.0	0.603	0.860
C18:3*n*-6	0.03	0.04	0.04	0.04	0.003	0.255
C18:2*t*9*t*12	0.14	0.14	0.13	0.15	0.011	0.794
C18:3*n*-3	0.46	0.56	0.57	0.58	0.035	0.099
C18:4*n*-3	0.01	0.02	0.02	0.02	0.002	0.100
C20:0	0.14	0.15	0.14	0.14	0.009	0.806
C20:1*c*11	0.29	0.24	0.24	0.25	0.021	0.282
C20:2*n*-6	1.09	1.21	1.13	1.04	0.085	0.564
C20:3*n*-6	1.13	1.04	1.03	1.11	0.095	0.843
C20:4*n*-6	11.7	13.1	12.1	11.5	1.034	0.695
C20:3*n*-3	0.08^a^	0.12^b^	0.12^b^	0.11^ab^	0.009	0.032
C20:5*n*-3	0.22	0.28	0.30	0.29	0.022	0.065
C22:0	0.07	0.07	0.07	0.07	0.005	0.973
C22:1*n*-9	0.01	0.01	0.01	0.01	0.002	0.589
C22:2*n*-6	0.015	0.012	0.016	0.012	0.002	0.316
C22:5*n*-3	1.24	1.18	1.07	1.02	0.111	0.481
C22:6*n*-3	1.95	3.02	2.79	2.63	0.324	0.130
Others	3.56	2.82	3.35	2.98	0.343	0.426
***Partial sums of FA, g/100 g FA***						
SFA^1^	43.3	42.8	43.8	43.1	0.568	0.659
MUFA^2^	15.2	12.4	12.8	14.8	1.785	0.601
PUFA^3^	38.1	41.2	39.9	38.5	1.932	0.667
*n*-6 PUFA^4^	34.0	35.9	34.9	33.7	1.594	0.773
*n*-3 PUFA^5^	3.97	5.18	4.87	4.64	0.396	0.191
***Ratios of FA***						
PUFA/SFA	0.88	0.97	0.92	0.90	0.053	0.672
*n*-6/*n*-3	8.79^a^	7.22^b^	7.38^ab^	7.57^ab^	0.382	0.025

The broilers were fed: (1) a corn-soybean based diet (Control); (2) the based diet plus 10% *C. vulgaris* (CH); (3) diet 2 supplemented with 0.005% Rovabio^®^ Excel AP; and (4) diet 2 supplemented with 0.01% of a pre-selected four-CAZyme mixture (CHM)

SEM - standard error of the mean; FA - fatty acids; DMA - dimethylacetal; SFA - saturated fatty acids; MUFA - monounsaturated fatty acids; PUFA - polyunsaturated fatty acids

^1^ Sum (C10:0, C12:0, C14:0, C15:0, C16:0, C17:0, C18:0, C20:0, C22:0)

^2^ Sum (C14:1*c*9, C16:1*c*7, C16:1*c*9, C17:1*c*9, C17:1*c*10, C18:1*c*9, C18:1*c*11, C20:1*c*11, C22:1*n*-9)

^3^ Sum (C18:2*n*-6, C18:2*t*9*t*12, C18:3*n*-6, C18:3*n*-3, C18:4*n*-3, C20:2*n*-6, C20:3*n*-6, C20:4*n*-6, C20:3*n*-3, C20:5*n*-3, C22:5*n*-3, C22:6*n*-3)

^4^ Sum (C18:2*n*-6, C18:3*n*-6, C20:2*n*-6, C20:3*n*-6, C20:4*n*-6)

^5^ Sum (C18:3*n*-3, C18:4*n*-3, C20:3*n*-3, C20:5*n*-3, C22:5*n*-3, C22:6*n*-3)

^a.b^ Different superscripts within a row indicate a significant difference (*p* < 0.05)

### Hepatic tocopherols and pigments

Hepatic vitamin E homologues and pigments of broilers fed *C. vulgaris*, individually or combined with exogenous CAZymes, are presented in Table 4. Although α-tocopherol was unchanged by the experimental diets, γ-tocopherol was consistently decreased in broilers fed *C. vulgaris*, regardless the presence of CAZymes (*p* < 0.001). In contrast, β-carotene increased with CH and CHR diets compared to the control diet (*p* < 0.001). Chlorophyll-*a* increased with CHM relative to the control and CH diets (*p* = 0.011). In addition, broilers fed microalga diets had higher total carotenoids and total chlorophylls plus carotenoids than broilers fed the control diet (*p* < 0.001).

**Table 4:** Effect of experimental diets on α-tocopherol, γ-tocopherol and pigments of liver from broilers

Item	Control	CH	CHR	CHM	SEM	*p*-value
***Diterpene profile, µg/g***						
α-Tocopherol	6.66	6.07	5.43	4.91	0.626	0.199
γ-Tocopherol	1.26^b^	0.822^a^	0.833^a^	0.840^a^	0.070	< 0.0001
***Pigments, µg/g***						
β-Carotene	0.977^a^	3.66^c^	3.15^bc^	2.11^ab^	0.388	< 0.0001
Chlorophyll-*a*^1^	2.44^a^	2.12^a^	3.66^ab^	4.21^b^	0.477	0.011
Chlorophyll-*b*^2^	4.94	3.36	6.12	5.68	0.897	0.161
Total chlorophylls^3^	7.33	5.49	9.78	9.67	1.38	0.102
Total carotenoids^4^	2.14^a^	10.1^b^	10.5^b^	8.49^b^	0.860	< 0.001
Total chlorophylls + Carotenoids^5^	9.48^a^	15.7^b^	20.2^b^	18.2^b^	1.65	< 0.001

The broilers were fed: (1) a corn-soybean based diet (Control); (2) the based diet plus 10 % *C. vulgaris* (CH); (3) diet 2 supplemented with 0.005 % Rovabio® Excel AP; and (4) diet 2 supplemented with 0.01 % of a pre-selected four-CAZyme mixture (CHM)

SEM - standard error of the mean

^1^ Ca = 11.24 × A_662_ − 2.04 × A_645_

^2^ Cb = 0.13 × A_645_ − 4.19 × A_662_

^3^ Ca + b = 7.05 × A_662_ + 18.09 × A_645_

^4^ Cx + c = (1000 × A_470_ − 1.90 × Ca − 63.14 × Cb) /214

^5^ (Ca + b) + (Cx + c)

^a,b,c^ Mean values within a row with unlike superscript letters are significantly different (*p* < 0·05)

### Principal component analysis

**Fig. 1:**
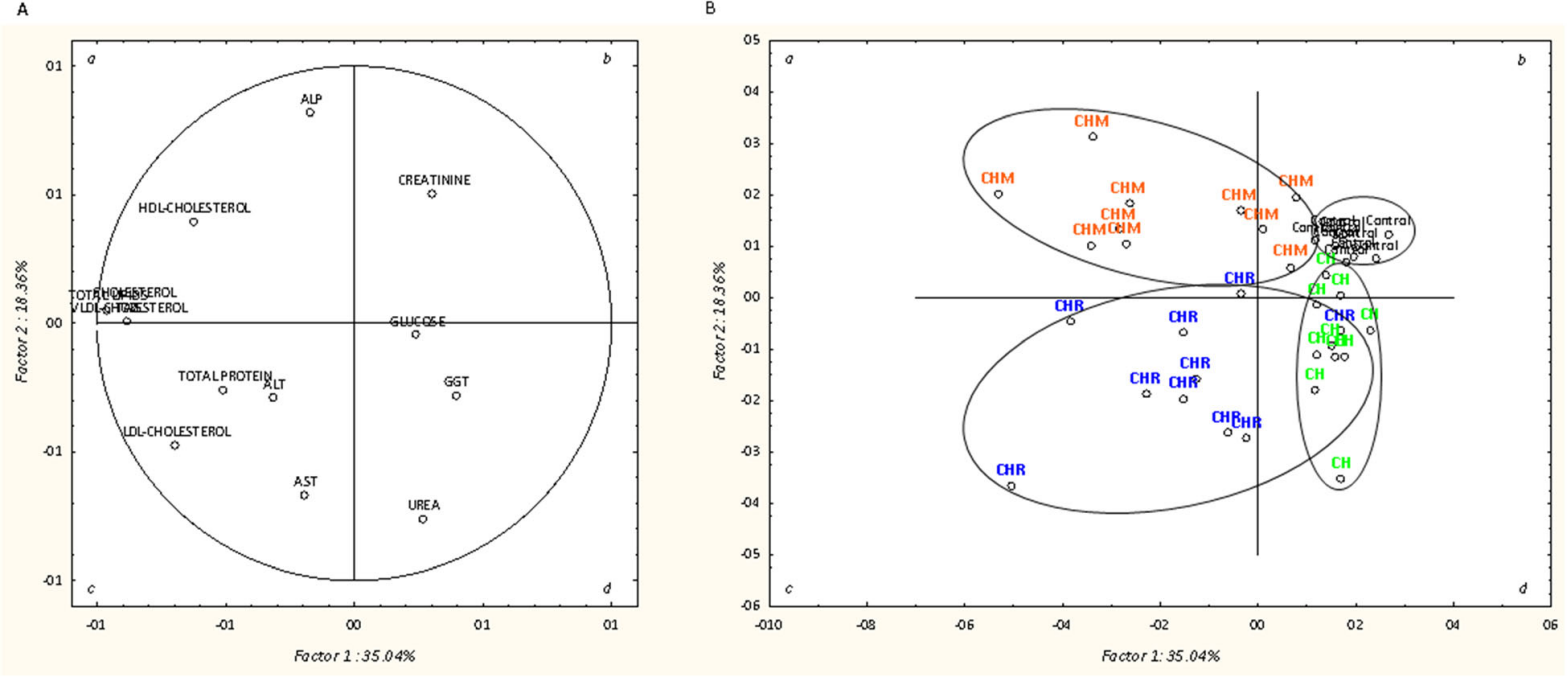
Loading plot of the first and second principal components of the pooled data (**A**) and component score vectors (**B**) using plasma metabolites from broilers fed *Chlorella vulgaris*, individually and combined with exogenous CAZymes. Dietary treatments: corn-soybean meal based diet (control); based diet with 10% of *C. vulgaris* (CH); based diet with 10% of *C. vulgaris* supplemented with 0.005% Rovabio^®^ Excel AP (CHR); based diet with 10% of *C. vulgaris* supplemented with 0.01% of a pre-selected four-CAZyme mixture (CHM)

Principal component analysis (PCA) was performed to evaluate the relationship of plasma metabolites and hepatic lipid composition of broilers fed the four experimental diets. Hepatic parameters had no relationship (see Supplementary Fig. 1) using this discriminant analysis. Hence, Fig. 1 A shows only the PCA of plasma metabolites to describe the variability of the pooled data into two dimensions. The first two discriminant factors explained about 53.4% of total variability, with 35.0 % for factor 1 and 18.4% for factor 2. The loadings for the first two factors obtained for each variable are presented in Table 5. Plasma metabolites with the highest discriminant power were total lipids, TAG, VLDL-cholesterol and total cholesterol, on the factor 1, and ALP, urea and AST, on the factor 2. The PCA model revealed a good separation of the experimental groups, which are located in different quadrants (Fig. 1B). The control group was set well aggregated in quadrant *b* and CH group in quadrant *d*. The other groups fed microalga-based diets supplemented with exogenous CAZymes, were located more dispersed in quadrants *c* (CHR) and *a* (CHM).

**Table 5:** Loadings for the first two principal components

Variables	Factor 1	Factor 2
Total lipids	-0.96	0.05
TAG	-0.88	0.01
Total cholesterol	-0.86	0.07
HDL-cholesterol	-0.63	0.40
LDL-cholesterol	-0.70	-0.48
VLDL-cholesterol	-0.88	0.01
Glucose	0.24	-0.05
Urea	0.26	-0.76
Creatinine	0.31	0.51
Total protein	-0.51	-0.26
ALT	-0.31	-0.29
AST	-0.19	-0.67
ALP	-0.17	0.82
GGT	0.39	-0.28

TAG - triacylglycerols; HDL - high-density lipoproteins; LDL - low-density lipoproteins; VLDL - very low-density lipoproteins; ALT - alanine aminotransferase (EC 2.6.1.2); AST - aspartate aminotransferase (E.C. 2.6.1.1); ALP - alkaline phosphatase (EC 3.1.3.1); GGT - gamma-glutamyltransferase (EC 2.3.2.13)

## Discussion

In the present study, the dietary incorporation of (10% *C. vulgaris*, supplemented or not with the two feed CAZyme mixtures, Rovabio^®^ and the four-CAZyme mixture described by Coelho et al. [17], has no significant effects on productive parameters and, therefore, does not compromise growth performance of broilers. These findings partially agree with the literature because, so far, most of the studies in poultry nutrition are focused in the use of *C. vulgaris* as supplement (< 2% in diet) [2, 23]. Previous reports observed that low incorporation levels (0.07–1.25%) of *Chlorella* in broilers promotes a decrease of FCR with no influence in ADG [24, 25]]. Similarly, Dlouha et al. [26] and Kang et al. [27] described a positive effect on ADG without changes on feed/gain ratio and FCR with supplementation of *Chlorella sp.* in broiler chicken diets during 42 and 28 days, respectively. Thus, it seems that the level of *C. vulgaris* inclusion in the diet and the duration of the experimental trial impact broilers performance. Herein, the supplementation of 10 % *C. vulgaris* with the two exogenous CAZymes does not seem to be necessary in the response of broilers to productive parameters.

Plasma biochemical parameters are a useful tool for assessing metabolic changes in organs and tissues. Even though plasma lipid profile was largely affected by the dietary treatments, our results does not confirm the findings of Kotrbáček et al. [28], who reported that dietary *Chlorella* biomass did not affect plasma TAG and cholesterol concentrations in laying hens. Likewise, An et al. [29] found that blood parameters, including albumin, total protein, AST, total cholesterol, HDL cholesterol and triacylglycerols, were not altered by dietary treatments in broiler chickens. Lately, Abdelnour et al. [23] showed that blood total protein and HDL-cholesterol of broilers can be positively affected by addition of low amounts (0.5-1.0% of the diet) of *C. vulgaris* biomass to feed. Here, the concentration of total protein also increased with the incorporation of *C. vulgaris* at high-level (10 %) in broiler diets, individually or combined with both mixtures of exogenous CAZymes. However, the increment in cholesterol transport of LDL promoted by this microalga, and even more in association with exogenous CAZymes, was not countered by reverse cholesterol transport of HDL, increasing the ratio total cholesterol/HDL-cholesterol. Moreover, total lipids, total cholesterol and VLDL-cholesterol were higher in broilers fed *C. vulgaris* supplemented with both exogenous CAZymes. Data suggest that *C. vulgaris*, at this high level (10%), might enhance the intestinal absorption of dietary lipids. Thus, the present study does not corroborates the well-established cholesterol- and lipid-lowering properties of *Chorella* [30]. Although the mechanism of the hypocholesterolemic effect are still unclear, it seems that *Chlorella* enhances cholesterol catabolism through the up-regulation of hepatic cholesterol 7α-hydroxylase expression [31]. In addition, *Chlorella* may inhibit the intestinal absorption of excess cholesterol from the diet and to enhance faecal steroid excretion, and thus, preventing hypercholesterolemia [32]. Animal and human trials have been shown an ameliorative effect on plasma lipid profiles [33, 34] upon supplementation of *Chlorella*, which can be ascribed to a decrease in intestinal tract fat absorption [35]. Zheng et al. [20] found lower TAG levels in laying hens supplemented with fermented *C. vulgaris.* The former authors hypothesised that the reason for the decreased plasma TAG concentration of laying hens was the inhibition of hepatic fatty acid synthesis and triacylglycerol production. Previous studies have shown that the nutritional composition of *C. vulgaris* may contribute to their effects on mitigating metabolic alterations through different mechanisms [36, 37]. Several algae-derived bioactive compounds, like lipids, antioxidants, pigments, vitamins and polysaccharides are known to have beneficial effects on human and animal health [38–40]. In contrast, here, data suggest that both feed carbohydrate-degrading enzymes (Rovabio^®^ and the pre-selected four-CAZyme mixture) were effective to hydrolyse *C. vulgaris* cell wall polysaccharides that changed fibre gut profile, thus reducing its anti-hyperlipidaemic activity. To date, animal studies have investigated the potential hypoglycaemic effect of *Chlorella* and the mechanisms by which *Chlorella* might exert protection against diabetes [41, 42]. In the current study, glucose was found decreased with *C. vulgaris* when combined with the four-CAZyme mixture, suggesting a positive effect on glycemia homeostasis. The aforementioned studies [41, 42] reported that the intake of low levels of *Chlorella*, in normal and induced-diabetic mice and insulin resistant rats, respectively, might lower the plasma glucose but affects the insulin secretion capacity very slightly or not at all. Therefore, further experiments are required to clarify the hypoglycaemic effect of *Chlorella* and to elucidate the effective doses that are responsible for the positive effect on insulin sensitivity. The antidiabetic effect of *Chlorella* has been linked to the action of *Chlorella*-derived components, including polysaccharides [43, 44]. Also, *C. vulgaris* has been reported to have antioxidant properties and even to aid detoxification. It is well established that hepatocytes are in the frontline against oxidative damage [45]. Even though growing evidence from animal and human studies suggests that *C. vulgaris* can be a promising hepatoprotective agent, the results are still controversial, as recently reviewed by Yarmohammadi et al. [46]. Herein, none of the aminotransferases enzymes (AST and ALT) was affected by dietary inclusion of 10 % *C. vulgaris*, supplemented or not with both exogenous CAZymes. In contrast, Abdelnour et al. [23] reported a decrease of 23 % in the levels of liver enzymes in broilers supplemented with dietary *C. vulgaris* compared to the control birds. In the present study, ALP and GGT activities changed in the opposite direction, while ALP levels increased in broilers fed microalga supplemented with the four-CAZyme mixture, the GGT levels significantly decreased. Regarding indicators of renal function, creatinine kept unchanged whereas urea reached the highest value with *C. vulgaris* incorporation. The discrepancy in response to hepatic and renal biomarkers in different trials could be partly ascribed to the dosage and source of microalga, as well as experimental period duration and conditions. Overall, plasma biochemical parameters enabled broilers to be assigned into their experimental diets with good accuracy, which are well distributed by the four quadrants, as shown by the discriminant analysis.

Liver is the hub of cholesterol synthesis and fatty acid oxidation. Moreover, *de novo* lipogenesis occurs essentially in both liver and adipose tissue [47]. Hepatic total lipids and total cholesterol concentrations as well as the majority of individual fatty acids identified were not affected by the incorporation of 10% *C. vulgaris* in diets nor by the supplementation with the exogenous CAZymes. However, C16:1*c*7 and C20:3*n*-3 increased about 1.7-fold and 1.5-fold, respectively, in broilers fed *C. vulgaris* compared with the control group. In addition, the *n*-6/*n*-3 ratio showed a significant reduction, of about 18%, in microalga-fed animals compared to the control group, which indicates that CH diet, in general, promoted an increase in the concentration of *n*-3 PUFA in broiler liver. Our results agree with those conducted by Zheng et al. [20], who analysed the effect of dietary fermented *C. vulgaris* on growth performance, liver lipids and intestinal microflora of laying hens. The former authors found only a significant effect in triacylglycerols, without no differences for cholesterol and phospholipids. Later, Gatrell and colleagues [48] using different levels of incorporation of defatted green microalga *Nannochloropsis oceanica* biomass observed an increase of C20:5*n*-3 and C22:6*n*-3 fatty acids, as well as in the sum of *n*-3 PUFA, accompanied with a decrease of the *n*-6/*n*-3 ratio in liver of broilers. It is well known that *Nannochloropsis oceanica* presents a superior concentration of *n*-3 PUFA when compared with *C. vulgaris*. Hence, defatted green microalga *Nannochloropsis oceanica* promoted more extensive variations in liver lipids than those obtained herein by dietary incorporation of *C. vulgaris* in broilers. Tao et al. [49] also documented an increase of *n*-3 PUFA and a decrease of the *n*-6/*n*-3 ratio in liver with the inclusion of 10% of defatted *N. oceanica* biomass in chicken diet. This enrichment of liver in *n*-3 PUFA has been associated with the downregulation of PUFA oxidation–related gene expression, attenuated lipid peroxidation and enhanced antioxidant activities [49].

The influence of dietary incorporation of *C. vulgaris*, supplemented or not with exogenous CAZymes, on hepatic levels of vitamin E and pigments was also explored. α-Tocopherol was the major vitamin E homologue in all groups fed with the experimental diets, while γ-tocopherol was present at lower concentrations, which strongly agrees with diet composition. Tao et al. [49] also documented no changes on hepatic α-tocopherol levels of broilers through the incorporation of 10% defatted *N. oceanica*, probably due to similar vitamin E content in both microalgae [50, 51]. *C. vulgaris* is also rich in pigments, such as chlorophylls and carotenoids, due to the photosynthetic pathway [52, 53]. β-carotene content, a precursor of vitamin A, increased about 3.7-fold in liver of broilers fed *C. vulgaris* alone, whereas total carotenoids increased about 4.5-fold in all microalga groups. These results agree with previous researches, which observed a consistent increase of lutein and total carotenoids in liver of laying hens fed with conventional or lutein-enriched *Chlorella* [54, 55]. Although the dietary inclusion of 10% *C. vulgaris* did not allow an increase in hepatic vitamin E, the raise of β-carotene and total carotenoids contents in liver is a key indicator of its bioavailability from diets. Hence, *C. vulgaris* is an excellent source of antioxidant compounds, like α-tocopherol and carotenoids, which are known to counterbalance oxidative stress and promote animal health [56].

## Conclusions

Collectively, data indicate that *C. vulgaris* incorporated as a feed ingredient (10%) in broiler diets improves liver composition but negatively affects systemic lipemia, without impairing, in general, animal health and growth performance. Moreover, the supplementation of diets with the exogenous CAZymes are no needed at this inclusion level of *C. vulgaris*. Although these results indicate the viability of *C. vulgaris* as feedstock in poultry nutrition, further experiments are required to confirm these findings under different experimental conditions, including other percentages of *C. vulgaris* incorporation in the diet, in order to determine the minimal effective dose for a positive effect on biochemical metabolites and lipid metabolism. Future work should also elucidate the molecular mechanisms involved in lipid metabolic changes.

## Methods

### Recombinant four-CAZyme mixture production

The pre-selected recombinant four-CAZyme mixture is composed by exo-β-glucosaminidase, alginate lyase, peptidoglycan N-acetylmuramic acid deacetylase and lysozyme, which in a concentration of 20 mg/L exhibits 1.21 g/L of reducing sugars released upon a 20 g/L of *C. vulgaris* suspension as subtract [17]. The genes encoding the four recombinant CAZymes, which composed the enzyme mixture, were cloned using the procedure described by Coelho et al. [17]. Succinctly, the generated recombinant plasmids were used to transform BL21 *Escherichia coli* cells that were grown on Luria-Bertani media until reach the mid exponential phase (0.4–0.6 of absorbance at λ = 595 nm). In order to induce recombinant gene expression, isopropyl β-d-thiogalactoside was added. The induction of protein expression occurred with incubation of BL21 cells overnight and, after ultrasonication of cells, centrifugation and freeze dried, the four-CAZyme protein extracts were mixed in equivalent weight amounts at a final level of 0.01%.

### Animals, feeding protocol and sampling

 The experimental procedures were carried out at the facilities of Instituto Superior de Agronomia (ISA, Universidade de Lisboa), reviewed by the Ethics Commission of CIISA (FMV) and approved by the Animal Care Committee of the National Veterinary Authority (DGAV, Portugal), following the ARRIVE guidelines and the European Union legislation (2010/63/EU Directive). One hundred and twenty Ross 308 male birds were raised in 40 wired-floor cages. Each cage was 66 × 66 cm. All birds were kept in a thermostatically controlled room with constant light. Environmental temperature was monitored continuously, which gradually decrease from 31 °C (day 0) to 21 °C (day 22) and remained constant until the end of the trial. The experimental design was performed with 10 replicate pens per treatment, with 3 birds per pen. Before the beginning of the trial, birds received a corn-based diet during 21 days. After an acclimation period, one of the four isocaloric and isonitrogenous diets were randomly allocated to each pen: (1) corn-soybean meal based diet (control); (2) based diet with 10% of *C. vulgaris* supplied by Allmicroalgae (Natural Products, Portugal) (CH); (3) diet 2 with 10% of *C. vulgaris* supplemented with 0.005% of the commercial CAZyme cocktail Rovabio^®^ Excel AP from Adisseo (Antony, France), containing predominantly endo–1,4-β-xylanase 22,000 viscosity unit/g and endo-1,3(4)-β-glucanase 30,000 viscosity unit/g (CHR); and (4) diet 2 with 10% of *C. vulgaris* supplemented with 0.01% of a pre-selected four-CAZyme mixture, containing exo-β-glucosaminidase, alginate lyase, peptidoglycan N-acetylmuramic acid deacetylase and lysozyme, as mentioned above (CHM). The experimental period lasted from day 21 to day 35. Table 6 shows the ingredients of the experimental diets. During the experiment, feed was provided daily and birds were weighed weekly. ADFI, ADG and FCR were determined for animal performance evaluation. After 35 days of trial, one bird per experimental unit was euthanised using electrical stunning followed by exsanguination, according to commercial abattoirs standard procedures. Blood samples were collected from the jugular vein and centrifuged at 1500 *g* for 15 min to obtain plasma. Liver samples were vacuum packed and stored at -20 °C, until total lipids, total cholesterol, fatty acid composition, pigments and diterpene profile analyses.

**Table 6:** Ingredients and additives of the experimental diets (% as fed basis)

	Experimental diets			
**Ingredients**	**Control**	**CH**	**CHR**	**CHM**
Corn	56.0	55.5	55.5	55.5
Soybean meal	37.0	26.5	26.5	26.5
Soybean oil	3.60	4.14	4.14	4.14
Sodium chloride	0.330	0.330	0.330	0.330
Calcium carbonate	1.06	1.00	1.00	1.00
Dicalcium phosphate	1.44	1.50	1.50	1.50
DL-Methionine	0.280	0.360	0.360	0.360
L-Lysine	0.000	0.370	0.370	0.370
Vitamin-mineral premix^1^	0.300	0.300	0.300	0.300
*Chlorella vulgaris* powder	-	10.0	10.0	10.0
Rovabio^®^ Excel AP	-	-	0.005	-
Mix of 4 CAZymes	-	-	-	0.010

The broilers were fed: (1) a corn-soybean based diet (Control); (2) the based diet plus 10 % *C. vulgaris* (CH); (3) diet 2 supplemented with 0.005 % Rovabio^®^ Excel AP; and (4) diet 2 supplemented with 0.01 % of a pre-selected four-CAZyme mixture (CHM)

^1^ Premix provided the following nutrients per kg of diet: pantothenic acid 10 mg, vitamin D_3_ 2400 IU, cyanocobalamin 0.02 mg, folic acid 1 mg, vitamin K_3_ 2 mg, nicotinic acid 25 mg; vitamin B_6_ 2 mg, vitamin A 10,000 UI, vitamin B_1_ 2 mg, vitamin E 30 mg, vitamin B_2_ 4 mg, Cu 8 mg, Fe 50 mg, I 0.7 mg, Mn 60 mg, Se 0.18 mg, Zn 40 mg

### ***C. vulgaris*** and experimental diets analyses

The proximate composition of *C. vulgaris* and experimental diets was analysed according to AOAC [57] methods. Dry matter (DM) was calculated from samples dried at 103 °C until constant weight. Crude protein of microalga and diets was determined by the Kjeldahl method using the nitrogen (N) content and the factor 6.25. The ash content and crude fat of samples were determined through the AOAC method 942.05 [57] and by automatic Soxhlet extraction with petroleum ether (Gerhardt Analytical Systems, Königswinter, Germany), respectively. Gross energy was determined by the complete combustion of samples in an adiabatic bomb calorimeter (Parr 1261, Parr Instrument Company, Moline, IL, USA).

Fatty acid methyl esters (FAME) composition of *C. vulgaris* and experimental diets was analysed by gas chromatography, after extraction and acid transesterification, using heneicosaenoic acid (C21:0) methyl ester as the internal standard. The diterpene profile of samples was determined by HPLC according to Prates et al. [58]. The quantification of pigments in samples was performed as described by Teimouri et al. [59], with slight modifications of Pestana et al. [60] using the equations of Hynstova et al. [61]. Table 7 presents the chemical composition of *C. vulgaris* and the experimental diets.

**Table 7:** Chemical composition of *Chlorella vulgaris* and experimental diets

	Microalga	Experimental Diets					
**Item**	***C. vulgaris***	**Control**		**CH**	**CHR**	**CHM**	
***Energy, kcal ME/kg as fed basis***	4586	4614		4627	4650	4615	
***Proximate composition, % as fed basis***							
**Dry matter**	93.1	89.0	89.6		89.3	86.4	
**Crude protein**	42.8	19.9	20.4		19.8	19.1	
**Crude fat**	8.73	6.59	7.56		7.63	7.41	
**Ash**	11.8	5.60	6.08		6.21	6.13	
***Estimated available limiting amino acid composition, % as fed basis***							
Arginine	3.89	1.42	1.08		1.08	1.08	
Histidine	0.65	0.55	0.43		0.43	0.43	
Isoleucine	1.26	1.04	0.78		0.78	0.78	
Leucine	2.45	1.81	1.44		1.44	1.44	
Lysine	2.63	1.13	1.11		1.11	1.11	
Methionine	0.45	0.60	0.61		0.61	0.61	
Phenylalanine	1.49	1.13	0.87		0.87	0.87	
Threonine	2.32	0.79	0.60		0.60	0.60	
Tryptophan	0.47	0.29	0.22		0.22	0.22	
Valine	3.52	1.12	0.86		0.86	0.86	
***Fatty acid profile, % total fatty acids***							
C14:0	1.10	0.10	0.14		0.14	0.19	
C16:0	17.2	12.4	12.6		12.6	13.2	
C16:1*c*9	3.90	0.09	0.95		0.98	1.15	
C18:0	3.00	2.77	2.83		2.81	2.99	
C18:1*c*9	11.7	21.6	22.1		22.6	23.2	
C18:1*c*11	0.00	1.35	1.63		1.58	1.81	
C18:2*n*-6	11.2	50.5	47.3		48.0	46.5	
C18:3*n*-3	10.1	5.24	5.47		5.58	5.62	
C20:0	0.20	0.33	0.32		0.33	0.33	
C20:1*c*11	0.10	0.22	0.25		0.23	0.27	
***Diterpene profile, µg/g***							
α-Tocopherol	19.2	10.5	42.2		12.4	20.2	
α-Tocotrienol	n.d.^+^	1.29	5.94		3.00	2.75	
β-Tocopherol	0.34	0.44	0.98		0.52	0.66	
γ-Tocopherol + β-tocotrienol	0.52	16.2	26.8		14.7	19.3	
γ-Tocotrienol	0.56	2.50	7.60		3.92	3.36	
δ-Tocopherol	0.36	2.00	4.44		2.79	2.90	
***Pigments, µg/g***							
β-Carotene	198	n.d.	83.6		37.3	45.1	
Chlorophyll *a*^1^	906	0.67	307		339	200	
Chlorophyll *b*^2^	171	0.90	96.3		104	40.0	
Total chlorophylls^3^	1077	1.57	404		444	240	
Total carotenoids^4^	228	3.61	102		108	47.7	
Total chlorophylls + Carotenoids^5^	1305	5.17	505		552	288	

The broilers were fed: (1) a corn-soybean based diet (Control); (2) the based diet plus 10 % *C. vulgaris* (CH); (3) diet 2 supplemented with 0.005 % Rovabio^®^ Excel AP; and (4) diet 2 supplemented with 0.01 % of a pre-selected four-CAZyme mixture (CHM)

DM - dry matter; ME - metabolized energy; n.d. - not detected

^+^ Co-eluted with α-tocopherol

^1^ Ca = 11.24 × A_662_ − 2.04 × A_645_

^2^ Cb = 0.13 × A_645_ − 4.19 × A_662_

^3^ Ca + b = 7.05 × A_662_ + 18.09 × A_645_

^4^ Cx + c = (1000 × A_470_ − 1.90 × Ca − 63.14 × Cb) /214

^5^ (Ca + b) + (Cx + c)

### Plasma biochemical assays

Biochemical analyses of the collected plasma were performed to determine lipid profile, glucose, urea, creatinine, total protein and liver function markers. The determination of glucose concentrations, triacylglycerols (TAG), urea, creatinine, total cholesterol, HDL-cholesterol, LDL-cholesterol and total protein, alanine aminotransferase (ALT, EC 2.6.1.2), aspartate aminotransferase (AST, EC 2.6.1.1), alkaline phosphatase (ALP, EC 3.1.3.1) and gamma-glutamyltransferase (GGT, EC 2.3.2.13) were performed in a Modular Hitachi Analytical System (Roche Diagnostics, Mannheim, Germany), through diagnostic kits (Roche Diagnostics). VLDL-cholesterol and total lipids were calculated by Friedewald et al. [22] and Covaci et al. [21] formulas, respectively.

### Analysis of total cholesterol and diterpenes in liver

The determination of total cholesterol, β-carotene and vitamin E homologues in liver samples was done using the protocol of Prates et al. [58]. After saponification and extraction with *n*-hexane, liver samples, in duplicate, were analysed by HPLC (Agilent 1100 Series, Agilent Technologies Inc., Palo Alto, CA, USA). Total cholesterol and β-carotene were detected by UV-Vis detection (λ = 202 nm and λ = 450 nm, respectively) while tocopherols and tocotrienols by fluorescence (excitation λ = 295 nm and emission λ = 325 nm). Quantification of total cholesterol and diterpenes in liver samples was performed using standard curves of peak area *versus* concentration.

### Analysis of pigments in liver

The determination of pigments was carried out as mentioned above for experimental diets, according to Teimouri et al. [59] with slight modifications by Pestana et al. [60]. The simultaneous extraction of pigments was performed by incubation of liver samples with acetone overnight (Merck KGaA, 249 Darmstadt, Germany). Then, samples were centrifuged and the absorbance of the supernatants were measured by UV-Vis spectrophotometry (Ultrospec 3100 pro, Amersham Biosciences, Little Chalfont, UK). The amount of pigments in liver samples was calculated as described by Hynstova et al. [61].

### Analysis of total lipids and fatty acid composition in liver

Total lipids were determined, in duplicate, gravimetrically from lyophilised (-60 °C and 2.0 hPa, lyophilizator Edwards Modulyo, Crawley, UK) liver samples according to Folch et al. [62]. Fatty acids were converted to FAME by sequential alkaline and acid transesterification and analysed by gas chromatography (HP7890A Hewlett-Packard, Avondale, PA) as described in Alfaia et al. [63]. Identification of FAME was based on the reference standard FAME mix 37 components (Supelco Inc.), which was confirmed by gas chromatography coupled with mass spectrometry using a GC-MS QP2010-Plus (Shimadzu, Kyoto, Japan). Heneicosanoic acid (C21:0) methyl ester was the internal standard used for the quantification of FAME. The fatty acids identified were expressed as the percentage of total fatty acids.

### Data analysis

The normal distribution and variance homogeneity were checked for all data using Shapiro–Wilk test and Levene’s test, respectively. Data were analysed by ANOVA using the PROC GLM of SAS software package (version 9.4; SAS Institute Inc., Cary, NC, USA). The dietary treatment (Control, CH, CHR and CHM) was the only source of variation (fixed effect). For feed intake and feed conversion ratio parameters, cage within each treatment was the experimental unit, whereas for blood and hepatic measurements, bird within each treatment was the experimental unit. Statistical differences among experimental diets were evaluated by least square means generated using the PDIFF option adjusted with Tukey-Kramer. The significance level was set at *p* < 0.05. In addition, a principal component analysis (PCA) was performed with the parameters measured in plasma and liver samples using the Statistica program (version 8.0; TIBCO software, Palo Alto, CA, USA).

## Supplementary information

Additional file 1 **Supplementary Figure 1. **Loading plot of the first and second principal components of the pooled data (**A**) and component score vectors (**B**) using hepatic parameters analysed from broilers fed *Chlorella vulgaris*, individually and combined with exogenous CAZymes. Dietary treatments: corn-soybean meal based diet (control); based diet with 10% of *C. vulgaris* (CH); based diet with 10% of *C. vulgaris *supplemented with 0.005% Rovabio® Excel AP (CHR); based diet with 10% of *C. vulgaris *supplemented with 0.01% of a pre-selected four-CAZyme mixture (CHM).

## Data Availability

All data generated during this study are included in this published article. The datasets generated during the current study are available from the corresponding author on demand.

## Abbreviations

ADFI: average daily feed intake
ADG: average daily gain
ALT: alanine aminotransferase
ALP: alkaline phosphatase
AST: aspartate aminotransferase
CAZymes: carbohydrate-active enzymes
CH: *Chlorella vulgaris*
FAME: fatty acid methyl esters
FCR: feed conversion ratio
GGT: gamma-glutamyltransferase
HDL: high-density lipoprotein cholesterol
LDL: low-density lipoprotein cholesterol
MUFA: monounsaturated fatty acids
PCA: principal component analysis
PUFA: polyunsaturated fatty acids
SFA: saturated fatty acids
TAG: triacylglycerols
VLDL: very-low-density lipoprotein cholesterol

## References

[1] Guaragni A, Boiago MM, Bottari NB, Morsch VM, Lopes TF, da Silva AS (2020). Feed supplementation with inulin on broiler performance and meat quality challenged with *Clostridium perfringens*: Infection and prebiotic impacts. Microb Pathog.

[2] Madeira MS, Cardoso C, Lopes PA, Coelho D, Afonso C, Bandarra NM, Prates JAM (2017). Microalgae as feed ingredients for livestock production and meat quality: a review. Livest Sci.

[3] Camacho F, Macedo A, Malcata F (2019). Potential industrial applications and commercialization of microalgae in the functional food and feed industries: a short review. Mar Drugs.

[4] Lum KK, Kim J, Lei XG (2013). Dual potential of microalgae as a sustainable biofuel feedstock and animal feed. J Anim Sci Biotechnol.

[5] Ryckebosch E, Bruneel C, Muylaert K, Foubert I (2012). Microalgae as an alternative source of omega-3 long chain polyunsaturated fatty acids. Lipid Technol.

[6] Draaisma RB, Wijffels RH, Slegers PE, Brentner LB, Roy A, Barbosa MJ (2013). Food commodities from microalgae. Curr Opin Biotechnol.

[7] Liu J, Chen F, Posten C, Feng Chen S (2014). Biology and Industrial Applications of Chlorella: Advances and Prospects. Microalgae Biotechnology. Advances in Biochemical Engineering/Biotechnology.

[8] Kotrbáček V, Doubek J, Doucha J (2015). The chlorococcalean alga *Chlorella* in animal nutrition: a review. J Appl Phycol.

[9] Acton Q. Cellular structures. In: Acton QA, editor. Advances in Research and Application. Scholarly Editions; 2013. p. 946.

[10] Baudelet PH, Ricochon G, Linder M, Muniglia L (2017). A new insight into cell walls of Chlorophyta. Algal Res.

[11] Teuling E, Wierenga PA, Agboola JO, Gruppen H, Schrama JW (2019). Cell wall disruption increases bioavailability of *Nannochloropsis gaditana* nutrients for juvenile Nile tilapia (*Oreochromis niloticus*). Aquaculture.

[12] Ravindran V, Son JH (2011). Feed enzyme technology: present status and future developments. Recent Pat Food Nutr Agric.

[13] Garron ML, Henrissat B (2019). The continuing expansion of CAZymes and their families. Curr Opin Chem Biol.

[14] Hashemi M, Seidavi A, Javandel F, Gamboa S (2017). Influence of non-starch polysaccharide-degrading enzymes on growth performance, blood parameters, and carcass quality of broilers fed corn or wheat/barley-based diets. Rev Colom Cienc Pecua.

[15] Cho HS, Oh YK, Park SC, Lee JW, Park JY (2013). Effects of enzymatic hydrolysis on lipid extraction from *Chlorella vulgaris*. Renew Energy.

[16] Phong WN, Show PL, Ling TC, Juan JC, Ng EP, Chang JS (2018). Mild cell disruption methods for bio-functional proteins recovery from microalgae - recent developments and future perspectives. Algal Res.

[17] Coelho D, Lopes PA, Cardoso V, Ponte P, Brás J, Madeira MS, Alfaia CM, Bandarra NM, Gerken HG, Fontes CMGA, Prates JAM (2019). Novel combination of feed enzymes to improve the degradation of *Chlorella vulgaris* recalcitrant cell wall. Sci Rep.

[18] Panahi Y, Darvishi B, Jowzi N, Beiraghdar F, Sahebkar A (2016). Chlorella vulgaris: a multifunctional dietary supplement with diverse medicinal properties. Curr Pharm Des.

[19] Fallah AA, Sarmast E, Habibian Dehkordi S, Engardeh J, Mahmoodnia L, Khaledifar A, Jafari T (2018). Effect of Chlorella supplementation on cardiovascular risk factors: a meta-analysis of randomized controlled trials. Clin Nutr.

[20] Zheng L, Oh ST, Jeon JY, Moon BH, Kwon HS, Lim SU, An BK, Kang CW (2012). The dietary effects of fermented Chlorella vulgaris (CBT®) on production performance, liver lipids and intestinal microflora in laying hens. Asian-Australas J Anim Sci..

[21] Covaci A, Voorspoels S, Thomsen C, van Bavel B, Neels H (2006). Evaluation of total lipids using enzymatic methods for the normalization of persistent organic pollutant levels in serum. Sci Total Environ.

[22] Friedewald WT, Levy RI, Fredrickson DS (1972). Estimation of the concentration of low-density lipoprotein cholesterol in plasma, without use of the preparative ultracentrifuge. Clin Chem.

[23] Abdelnour SA, Abd El-Hack ME, Arif M, Khafaga AF, Taha AE (2019). The application of the microalgae *Chlorella spp.* as a supplement in broiler feed. Worlds Poult Sci J.

[24] Rezvani M, Zaghari M, Moravej H (2012). A survey on *Chlorella vulgaris* effect’s on performance and cellular immunity in broilers. International J Agric Sci Res.

[25] Englmaierová M, Skrivan M, Bubancová I (2013). A comparison of lutein, spray-dried *Chlorella*, and synthetic carotenoids effects on yolk colour, oxidative stability, and reproductive performance of laying hens. Czech J Anim Sci.

[26] Dlouha G, Sevcikova S, Dokoupilova A, Zita L, Heindl J, Skrivan M (2008). Effect of dietary selenium sources on growth performance, breast muscle selenium, glutathione peroxidase activity and oxidative stability in broilers. Czech J Anim Sci.

[27] Kang HK, Salim HM, Akter N, Kim DW, Kim JH, Bang HT, Kim MJ, Na JC, Hwangbo J, Choi HC, Suh OS (2013). Effect of various forms of dietary *Chlorella* supplementation on growth performance, immune characteristics, and intestinal microflora population of broiler chickens. J Appl Poult Res.

[28] Kotrbáček V, Skrivan M, Kopecky J, Penkava O, Hudečková P, Uhríková I, Doubek J (2013). Retention of carotenoids in egg yolks of laying hens supplemented with heterotrophic CLV. Czech J Anim Sci.

[29] An BK, Kim KE, Jeon JY, Lee KW (2016). Effect of dried CLV *vulgaris* and CLV growth factor on growth performance, meat qualities and humoral immune responses in broiler chickens. Springer Plus.

[30] Cherng JY, Shih MF (2005). Preventing dyslipidemia by *Chlorella pyrenoidosa* in rats and hamsters after chronic high fat diet treatment. Life Sci.

[31] Shibata S, Hayakawa K, Egashira Y, Sanada H (2007). Hypocholesterolemic mechanism of *Chlorella*: *Chlorella* and its indigestible fraction enhance hepatic cholesterol catabolism through up-regulation of cholesterol 7α-hydroxylase in rats. Biosci Biotechnol Biochem.

[32] Lee HS, Park HJ, Kim MK (2008). Effect of *Chlorella vulgaris* on lipid metabolism in Wistar rats fed high fat diet. Nutr Res Pract.

[33] Shibata S, Oda K, Onodera-Masuoka N, Matsubara S, Kikuchi-Hayakawa H, Ishikawa F, Iwabuchi A, Sansawa H (2001). Hypocholesterolemic effect of indigestible fraction of *Chlorella regularis* in cholesterol-fed rats. J Nutr Sci Vitaminol.

[34] Ryu NH, Lim Y, Park JE, Kim J, Kim JY, Kwon SW, Kwon O (2014). Impact of daily Chlorella consumption on serum lipid and carotenoid profiles in mildly hypercholesterolemic adults: a double-blinded, randomized, placebo-controlled study. Nutr J.

[35] Sano T, Kumamoto Y, Kamiya N, Okuda M, Tanaka Y (1988). Effect of lipophilic extract on *Chlorella vulgaris* on alimentary hyperlipidemia in cholesterol-fed rats. Artery.

[36] de Jesus Raposo MF, de Morais AMMB, de Morais RMSC (2015). Marine polysaccharides from algae with potential biomedical applications. A review. Mar Drugs.

[37] Ramos-Romero S, Torrella JR, Pagès T, Viscor G, Torres JL (2021). Edible microalgae and their bioactive compounds in the prevention and treatment of metabolic alterations. Nutrients.

[38] Bito T, Okumura E, Fujishima M, Watanabe F (2020). Potential of *Chlorella* as a dietary supplement to promote human health. Nutrients.

[39] Barkia I, Saari N, Manning SR (2019). Microalgae for high-value products towards human health and nutrition. Mar Drugs.

[40] Liu WC, Guo Y, Zhihui Z, Jha R, Balasubramanian B (2020). Algae-derived polysaccharides promote growth performance by improving antioxidant capacity and intestinal barrier function in broiler chickens. Front Vet Sci.

[41] Cherng JY, Shih MF (2005). Potential hypoglycemic effects of *Chlorella* in streptozotocin-induced diabetic mice. Life Sci.

[42] Jeong H, Kwon HJ, Kim MK (2009). Hypoglycemic effect of *Chlorella vulgaris* intake in type 2 diabetic Goto-Kakizaki and normal Wistar rats. Nutr Res Pract.

[43] Tsai JS, Liu PY, Pan Sun B. A composition for reducing insulin resistance. Patent TWI487529B, 11 June 2015. p. 1–41.

[44] Wang P, Cui J. Preparing *Chlorella* polysaccharides for preventing and treating diabetes, and its application. Patent CH108164613A, 22 January 2018.

[45] Jadeja RN, Devkar RV, Nammi S. Oxidative stress in liver diseases: pathogenesis, prevention, and therapeutics. Oxid Med Cell Longev. 2017;8341286. 10.1155/2017/8341286 PMC5424478 28529677

[46] Yarmohammadi S, Hosseini-Ghatar R, Foshati S, Moradi M, Hemati N, Moradi S, Kermani MAJ, Farzaei MH, Khan H (2021). Effect of *Chlorella vulgaris* on liver function biomarkers: a systematic review and meta-analysis. Clin Nutr Res.

[47] Nafikov RA, Beitz DC (2007). Carbohydrate and lipid metabolism in farm animals. J Nutr.

[48] Gatrell SK, Kim J, Derksen TJ, O’Neil EV, Lei XG (2015). Creating ω-3 fatty-acid-enriched chicken using defatted green microalgal biomass. J Agric Food Chem.

[49] Tao L, Sun T, Magnuson AD, Qamar TR, Lei XG (2018). Defatted microalgae-mediated enrichment of *n*–3 polyunsaturated fatty acids in chicken muscle is not affected by dietary selenium, vitamin E, or corn oil. J Nutr.

[50] Andrade LM, Andrade C, Dias M, Nascimento C, Mendes M (2018). *Chlorella* and *Spirulina* microalgae as sources of functional foods, nutraceuticals, and food supplements; an overview. MOJ Food Process Technol.

[51] Zanella L, Vianello F (2020). Microalgae of the genus Nannochloropsis: chemical composition and functional implications for human nutrition. J Funct Foods.

[52] Batista AP, Gouveia L, Bandarra NM, Franco JM, Raymundo A (2013). Comparison of microalgal biomass profiles as novel functional ingredient for food products. Algal Res. 2013;2:164 – 73.

[53] Świątkiewicz S, Arczewska-Włosek A, Józefiak D (2015). Application of microalgae biomass in poultry nutrition. Worlds Poult Sci J.

[54] Leeson S, Caston L, Namkung AH (2007). Effect of dietary lutein and flax on performance, egg composition and liver status of laying hens. Can J Anim Sci.

[55] An BK, Jeon JY, Kang CW, Kim JM, Hwang JK (2014). The tissue distribution of lutein in laying hens fed lutein fortified *Chlorella* and production of chicken eggs enriched with lutein. Korean J Food Sci Anim Resour.

[56] Nabi F, Arain MA, Rajput N, Alagawany M, Soomro J, Umer M, Soomro F, Wang Z, Ye R, Liu J (2020). Health benefits of carotenoids and potential application in poultry industry: a review. J Anim Physiol Anim Nutr.

[57] AOAC. 2000. Official methods of analysis. Assoc. Offic. Anal. Chem. 17th ed. Arlington, VA, USA. AOAC. Official methods of analysis. 17th ed. Arlington: Assoc Offic Anal Chem. 2000.

[58] Prates JAM, Quaresma MAG, Bessa RJB, Fontes CMGA, Alfaia CMPM (2006). Simultaneous HPLC quantification of total cholesterol, tocopherols and [beta]-carotene in Barrosã-PDO veal. Food Chem.

[59] Teimouri M, Amirkolaie AK, Yeganeh S (2013). The effects of *Spirulina platensis* meal as feed supplement on growth performance and pigmentation of rainbow trout (*Oncorhynchus mykiss*). Aquaculture.

[60] Pestana JM, Puerta B, Santos H, Madeira MS, Alfaia CM, Lopes PA, Pinto RMA, Lemos JPC, Fontes CMGA, Lordelo MM, Prates JAM (2020). Impact of dietary incorporation of Spirulina (*Arthrospira platensis*) and exogenous enzymes on broiler performance, carcass traits, and meat quality. Poult Sci.

[61] Hynstova V, Sterbova D, Klejdus B, Hedbavny J, Huska D, Adam V (2018). Separation, identification and quantification of carotenoids and chlorophylls in dietary supplements containing *Chlorella vulgaris* and *Spirulina platensis* using high performance thin layer chromatography. J Pharm Biomed Anal.

[62] Folch J, Lees M, Stanley GHS (1957). A simple method for the isolation and purification of total lipids from animal tissues. J Biol Chem.

[63] Alfaia CM, Pestana JM, Rodrigues M, Coelho D, Aires MJ, Ribeiro DM, Major VT, Martins CF, Santos H, Lopes PA, Lemos JPC, Fontes CMGA, Lordelo MM, Prates JAM (2021). Influence of dietary *Chlorella vulgaris* and carbohydrate-active enzymes on growth performance, meat quality and lipid composition of broiler chickens. Poult Sci.

