# Supplementary figures and images for: Impact of dietary Chlorella vulgaris and carbohydrate-active enzymes incorporation on plasma metabolites and liver lipid composition of broilers
Source: BMC Vet Res. 2021 Jun 29;17:229. doi: 10.1186/s12917-021-02932-8 (PMC8243889; doi:10.1186/s12917-021-02932-8)

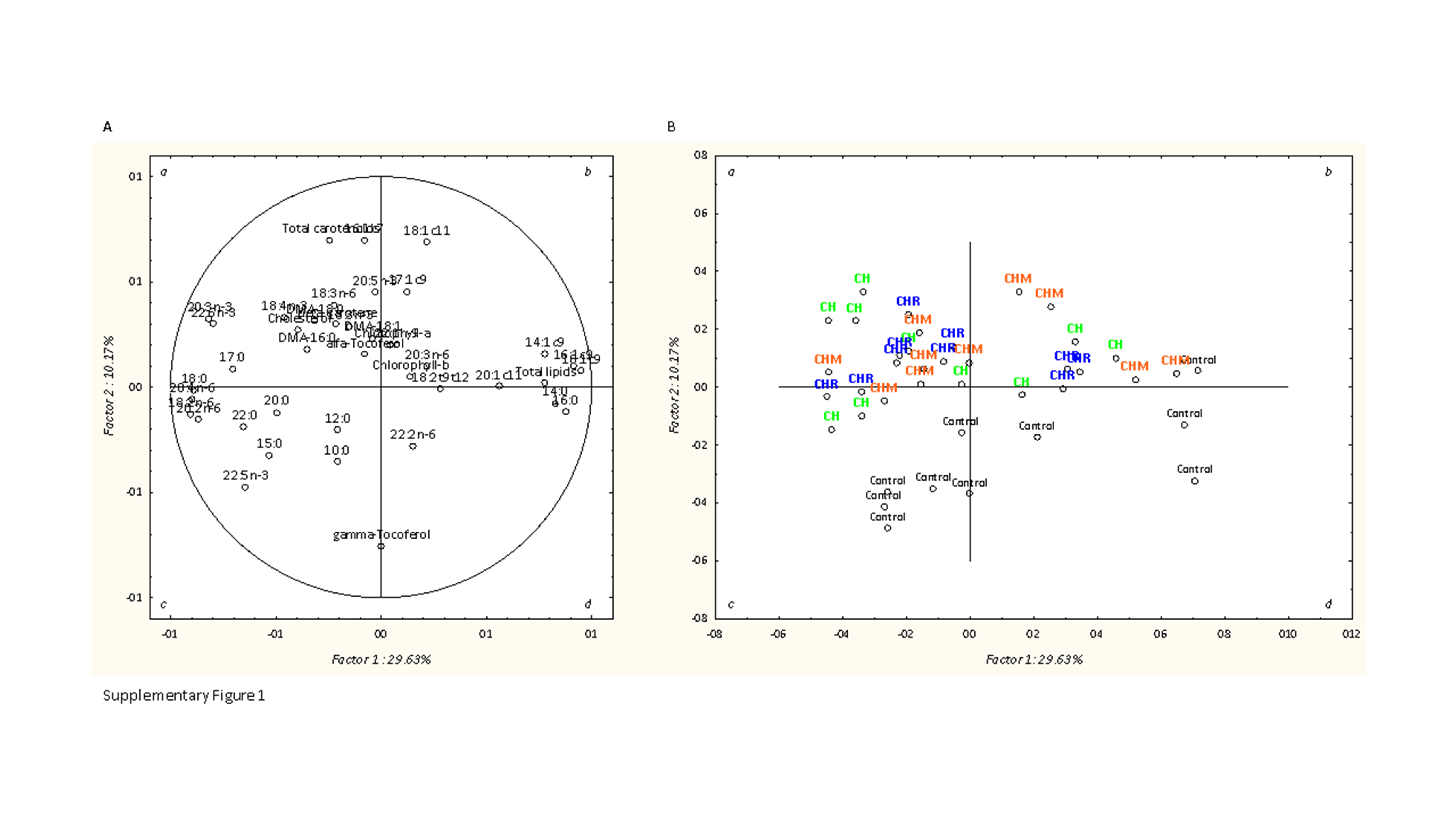

Supplement: Supplementary file 1 — Supplementary Figure 1. Loading plot of the first and second principal components of the pooled data (A) and component score vectors (B) using hepatic parameters analysed from broilers fed Chlorella vulgaris, individually and combined with exogenous CAZymes. Dietary treatments: corn-soybean meal based diet (control); based diet with 10% of C. vulgaris (CH); based diet with 10% of C. vulgaris supplemented with 0.005% Rovabio® Excel AP (CHR); based diet with 10% of C. vulgaris supplemented with 0.01% of a pre-selected four-CAZyme mixture (CHM). [file 12917_2021_2932_MOESM1_ESM.tif]
